# Adapting to a Changing World: Unraveling the Role of Man-Made Habitats as Alternative Feeding Areas for Slender-Billed Gull (*Chroicocephalus genei*)

**DOI:** 10.1371/journal.pone.0047551

**Published:** 2012-10-19

**Authors:** Francisco Ramírez, Joan Navarro, Isabel Afán, Keith A. Hobson, Antonio Delgado, Manuela G. Forero

**Affiliations:** 1 Estación Biológica de Doñana, Department de Biología de la Conservación, Sevilla, Spain; 2 Institut de Ciències del Mar, Barcelona, Spain; 3 Estación Biológica de Doñana, Laboratorio de SIG y Teledetección, Sevilla, Spain; 4 Environment Canada, Saskatoon, Saskatchewan, Canada; 5 Instituto Andaluz de Ciencias de la Tierra, Granada, Spain; University of Debrecen, Hungary

## Abstract

Current rates of wildlife habitat loss have placed increasing demands on managers to develop, validate and implement tools aimed at improving our ability to evaluate such impacts on wildlife. Here, we present a case study conducted at the Natural Area of Doñana (SW Spain) where remote sensing and stable isotope (δ^13^C, δ^15^N) analyses of individuals were combined to unravel (1) the effect of variations in availability of natural food resources (i.e. from natural marshes) on reproductive performance of a Slender-billed Gull (*Chroicocephalus genei*) population, and (2) the role of two adjacent, artificial systems (a fish farm and saltmines) as alternate anthropogenic feeding areas. Based on long-term (1983–2004) remote-sensing, we inferred the average extent of flooded area at the marshland (a proxy to natural resource availability) annually. Estimated flooded areas (ranging from extreme drought [ca. 151 ha, 1995] to high moisture [15,049 ha, 2004]) were positively related to reproductive success of gulls (estimated for the 1993–2004 period, and ranging from ca. 0 to 1.7 fledglings per breeding pairs), suggesting that habitat availability played a role in determining their reproductive performance. Based on blood δ^13^C and δ^15^N values of fledglings, 2001–2004, and a Bayesian isotopic mixing model, we conclude that saltmines acted as the main alternative foraging habitat for gulls, with relative contributions increasing as the extent of marshland decreased. Although adjacent, anthropogenic systems have been established as the preferred breeding sites for this gull population, dietary switches towards exploitation of alternative (anthropogenic) food resources negatively affected the reproductive output of this species, thus challenging the perception that these man-made systems are necessarily a reliable buffer against loss of natural feeding habitats. The methodology and results derived from this study could be extended to a large suite of threatened natural communities worldwide, thus providing a useful framework for management and conservation.

## Introduction

Human activities are impacting ecosystems globally to an unprecedented degree, thus leading to increasing rates of natural habitat loss [1]. Among natural ecosystems, coastal zones are of particular concern, since centuries of human influence have resulted in loss and degradation of more than 65% of natural habitats [2]. Consequently, more than 90% of species inhabiting these ecosystems are undergoing severe population declines [2], raising a grave conservation issue and placing increasing demands on managers to develop, validate and implement tools aimed at improving our ability to recognize and trace such environmental changes and evaluate their effects on free-living animals.

Simultaneously, the discipline of landscape ecology has expanded rapidly in recent years, supported by developments in spatial analysis techniques. In particular, remote sensing data is available from the last thirty years, thus providing valuable information on changes in habitat extent and heterogeneity at long spatial and temporal scales [3], [4], and resulting in a powerful tool for investigating the relationship between habitat loss or fragmentation on population dynamics of species [5]–[7]. However, species differ tremendously in their responses to environmental changes. In particular, the extent to which populations decrease following habitat loss strongly depends on the efficiency with which they can alter their foraging behavior in order to exploit alternative food resources [8], [9]. For example, several man-made structures, such as solar saltmines, fish farms or rice fields can provide complementary or alternate foraging areas for wild species naturally inhabiting and feeding on intertidal and estuarine systems when natural habitat loss occurs [9], [10]. Our ability to monitor spatiotemporal variations in the use of these alternate habitats by wildlife is therefore crucial for accurate assessments of the impact of habitat loss on wild populations.

Current approaches to resource use by wildlife based on naturally occurring stable isotope measurements of carbon (δ^13^C) and nitrogen (δ^15^N) provide an exceptional opportunity to quantify how wild species use anthropogenic habitats compared to the adjacent, natural environments, given that these differ in the isotopic composition of foodwebs (e.g. [11], [12]). Stable isotopes typically provide time-integrated information on animals’ diet by means of isotopic measurements of various consumer tissues [13], [14]. In addition, transforming isotopic information into dietary proportions of isotopically distinct dietary sources within a Bayesian or frequentist framework is now feasible through the use of multi-source isotope mixing models, thus allowing quantitative inference of nutrient pathways within natural and artificial systems [11], [15]. Isotopic approaches have therefore the potential for providing key insights into the actual role of man-made structures as alternative feeding areas for wild populations when natural habitat loss occurs.

**Figure 1:**
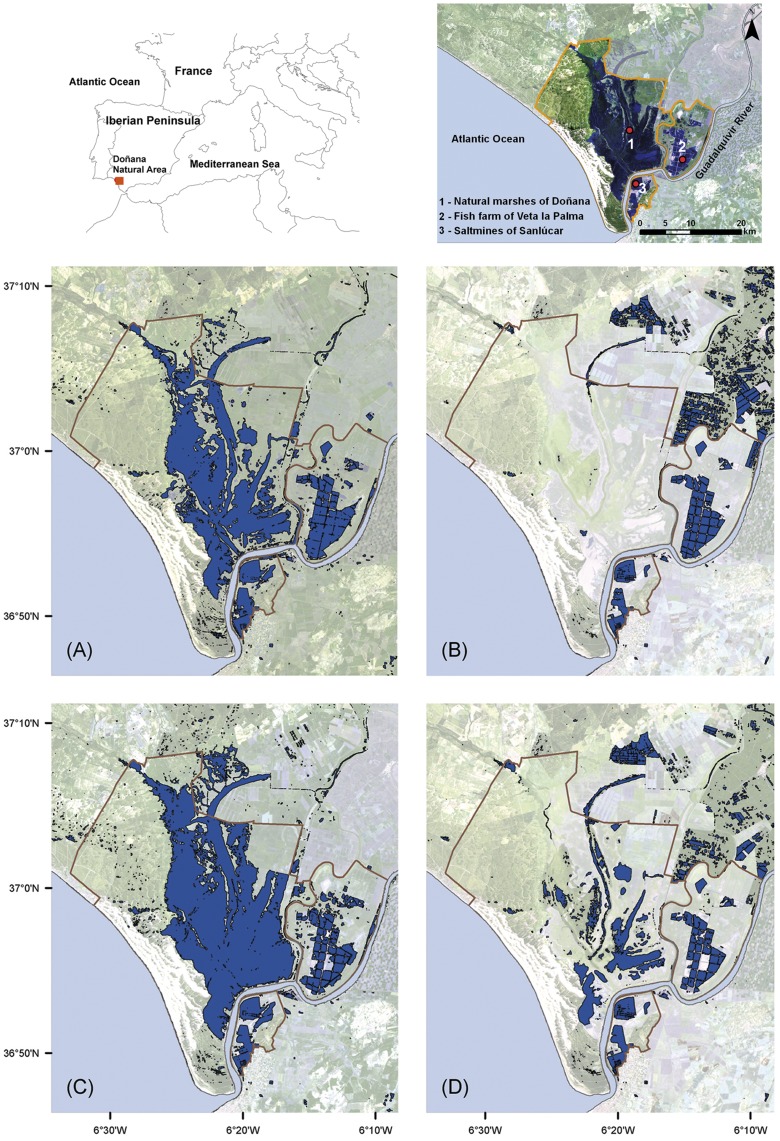
Temporal trends in the flooded area extent in the marshes of Doñana. Two examples of progressive inundation rates of the marshland of Doñana National Park in two different years. Inundation masks based on Landsat 7 (ETM sensor) images acquired on 29 April 2002 (A), 18 July 2002 (B), and Landsat 5 (TM sensor) images from 26 April 2004 (C) and 15 July 2004 (D). The Landsat false color composite images include bands in 7-5-3 (SWIR, Near Infrared, Red) combination. Upper figures show geographical location of the study zone and nesting colonies of Slender-billed Gull.

Here, we provide an instructive case study conducted at the Natural Area of Doñana (SW Spain, Figure 1) aimed at investigating the actual impact of temporal fluctuations in the extent of natural habitats on a wild avian population. Doñana is the largest protected coastal wetland in Western Europe, and natural marshes there constitute the main foraging habitat for most waterbirds and other avian inhabitants [16]–[18]. However, resource availability in this natural environment is strongly variable and depends on the extent of marshland, which changes both seasonally (commonly peaking in February-March and progressively decreasing thereafter up to near disappearance by July) and annually (spanning the whole continuum between extreme flood-years and extreme drought-years in which the marsh never floods). In contrast, two permanently flooded man-made systems nearby (the fish farm of Veta la Palma and the saltmines of Sanlúcar, Figure 1) provide waterbirds with two alternative and stable foraging habitats. This system (i.e. the combination of natural marshes, the fish-farm and saltmines) represents, then, a unique opportunity to investigate the impact of environmental changes affecting the extent and function of natural systems over wild populations and the role of man-made systems as alternative feeding habitats.

We used a long-term (1983–2004) remote sensing data set to infer inter-annual fluctuations in the extent of natural marshes, assumed to be a reliable surrogate of natural and local resource availability [18], and evaluate their effect over the reproductive performance of the Slender-billed Gull (*Chroicocephalus genei*) population breeding at the Natural Area of Doñana for the 1993–2004 period. Given that resource availability may constrain or influence relevant breeding parameters throughout the entire reproductive cycle: such as the likelihood of laying eggs or skipping reproduction, number and size of eggs laid, rate of hatching success, and number of nestlings raised to fledgling age (see [18]); we hypothesized that (1) inter-annual fluctuations in the extent of marshland, and therefore on natural resource availability, during different key periods (i.e. clutch production, incubation and chick-rearing periods) will result in variations in the reproductive performance of gulls; (2) individuals may cope with such variations in resource availability through dietary exploitation of the two adjacent, anthropogenic systems (i.e. the fish farm and saltmines). This second hypothesis was approached by using δ^13^C and δ^15^N values in tissues of fledglings and isotopic mixing models to reconstruct their use of various habitats for the 2001–2004 breeding period. Further insights into the actual role of such artificial systems as alternate feeding habitats for gulls were obtained by investigating the relationship between dietary estimates and reproductive performance (i.e. breeding success and fledgling body condition). To the best of our knowledge, this is the first study where remote sensing analyses of habitat availability and stable isotope approaches have been applied simultaneously for investigating the actual effect of habitat loss and fragmentation on population parameters. We argue that this approach provides an important advance in the way we tackle species conservation in a rapidly changing world.

## Methods

### Ethics Statement

The authors declare that all animals were handled in strict accordance with good animal practice as defined by the current European legislation, and all animal work was approved by the respective regional committees for scientific capture (“Consejería de Medio Ambiente de la Junta de Andalucía”, Sevilla, Spain). All necessary permits were obtained for the described field studies (provided by “Dirección General de Gestión del Medio Natural-Consejería de Medio Ambiente de la Junta de Andalucía” and “Dirección Natural de Espacios Naturales y Participación Ciudadana- Consejería de Medio Ambiente de la Junta de Andalucía”).

### Study Area

The study was conducted at Doñana National and Natural Park (i.e the Natural Area of Doñana), a protected wetland (50,000 ha) at the Guadalquivir River estuary (SW Spain, see Figure 1). The area was declared a National Park in 1969, and, afterwards, a World Heritage Site, Biosphere Reserve (UNESCO), Important Wetland Site under the Ramsar Convention (1982), Important Bird Area (IBA) and Nature 2000 site. Strong human-induced changes, mainly due to agricultural practices and water channel drainage, occurred during the 20^th^ Century leading to the current 27,000 ha of freshwater marshes within the National Park [19]. Natural marshes are annually flooded (between October and March) by winter rains, and then progressively dry up afterwards. Thus, the extent of the marshland radically varies both annually and seasonally according to the rainfall and climate regime [20]. Within the natural marshes, several flat islands, such as Veta de las Vaquiruelas, provide appropriate nesting sites for waterbirds. In the Natural Park, ca. 3,500 ha of the former marshland were transformed in 1992 for commercial fish farming. This private area, known as Veta la Palma (Figure 1), is formed by ca. 40 interconnected ponds with a permanent flooding regime that holds brackish water pumped from the Guadalquivir River estuary and contains re-vegetating islands for nesting waterbirds (see [16], [17], [21]). Within the limits of the Natural Park, ca. 3,000 ha are dominated by a commercial salt pan complex, the so-called saltmines of Sanlúcar (Figure 1). Salt production at this area goes back to the XV^th^ century, although latest modifications of this salt pan complex were carried out during the sixties. This man-made system includes several permanently flooded ponds of increasing salinities that ultimately leads to low-diversity communities mainly consisting of brine-shrimp [16]. The three areas included in this study (i.e. the natural marshes of the Doñana National Park, the fish farm of Veta la Palma and the saltmines of Sanlúcar) show, therefore, considerable differences in type of habitat and hydrological regimens and conditions that likely affect the availability of trophic resources and their accessibility to free-living animals.

### Studied Species

**Figure 2:**
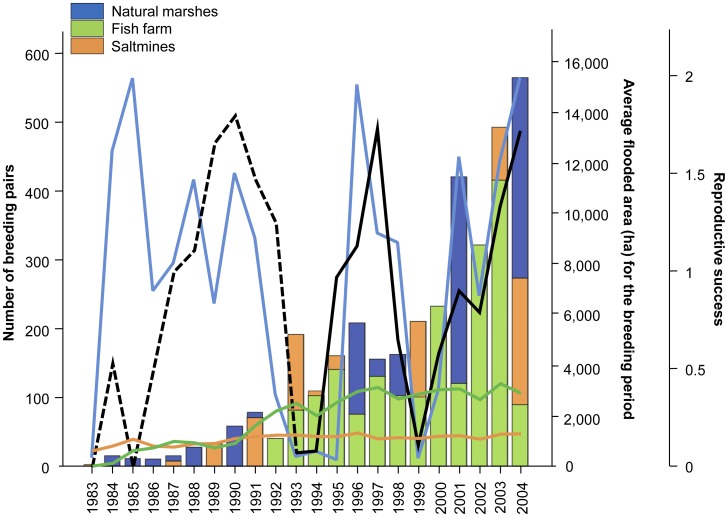
Linking natural resource availability with reproductive performance. Long-term (1983–2004) evolution of: bars =  number of breeding pairs for the Slender-billed Gull population breeding at the Natural Area of Doñana (split by breeding site); colored lines =  average extent of the flooded area for the natural marshes (blue), the fish farm (green; data for the 1983–1991 period refer to the flooded area of the former marshland that was transformed for commercial fish farming in 1992) and the saltmines (orange) during the Slender-billed gull breeding period (April-July); black line =  overall reproductive success of the Slender-billed gull population (dashed black line represents the estimated reproductive success before 1993, which have been estimated based on few and non systematic direct observations and have not been considered in the analyses, see Material and Methods).

The Slender-billed Gull is a medium-sized gull that breeds locally from Senegal and Mauritania, throughout the Mediterranean to Western India [22]. Doñana harbors an important breeding population of this species. From 1992 onward, the population has increased exponentially in parallel with the establishment of the commercial fish farm of Veta la Palma (Figure 2). For breeding, individuals select alternatively or simultaneously the above-mentioned three different areas (see Figures 1 and 2). Three different criteria made this gull species an ideal candidate for evaluating the effect of inter-annual fluctuations in the availability of natural food resources and the role of artificial systems as alternate feeding habitats: (1) individuals were expected to feed on resources both from natural and anthropogenic systems (previous dietary reports suggested this species to be mainly piscivorous, although individuals are commonly observed on saltmines consuming brine-shrimps, *Artemia spp*., [11], [23], [24]), with relative contributions presumably varying according to fluctuations in availability; (2) they tend to breed on isolated colonies in high densities [24], thus allowing accurate characterization of several population parameters (e.g. population size and reproductive success); and (3) the high-energy demanding phase of chick-rearing in this species (June–July) matches the period of maximum uncertainty in the extent of the flooded area at natural marshes, so that reproductive performance (measured as reproductive success and fledglings’ body condition) was expected to be particularly sensitive to fluctuations in the availability of natural resources.

### Fieldwork Procedures

Long-term (1983–2004) information on Slender-billed Gull population parameters, such as the total number of breeding pairs and fledglings has been recorded by six expert ornithologists of the Monitoring Team of Natural Processes of the Biological Station of Doñana. This information was initially (1983–1992) based on non-systematic direct observations. However, banding campaigns were implemented since 1993 and all breeding colonies located within this area were visited at least once per year matching up with the end of the chick-rearing period, thus allowing the accurate recording of the total number of nests and fledglings for each breeding site. Between 2001 and 2004, fledglings were also weighed (±10 g) and measured (maximum head length; ±1 mm) using a dynamometer and a digital caliper (head lengths for the 2001 breeding season are not available), respectively. Morphometric measurements were always taken by the same person thus avoiding any collector bias. In addition, from a subsample of fledglings randomly selected, 1 ml of blood was taken from the brachial vein for isotope (δ^13^C and δ^15^N) analyses (see Table 1 for sample sizes by year). Additionally, staff from the Monitoring Team carried out monthly censuses of Slender-billed Gulls for the 2000–2004 breeding period at the fish farm and saltmines (natural marshes were not visited as they are not easily accessible when flooded).

**Table 1:** Dietary reconstructions of Slender-billed Gull fledglings based on blood δ^13^C and δ^15^N values.

				Relative importance (%, 95th BCI)		
		δ^13^C (Mean ± SD ‰)	δ^15^N (Mean ± SD ‰)	SM	FF	NM
**2001**	NM (n = 14)	−16.38±1.25^2, 3^	14.52±0.75^2^	70.4–100	0–24.1	0–8.2
	FF (n = 18)	−15.32±0.50^1, 2^	14.64±0.26^1^	78.8–100	0–18.1	0–4.2
**2002**	FF (n = 56)	−15.62±0.89^1, 2^	13.62±0.50^2^	77.4–86.0	0–3.4	13.7–20.2
**2003**	FF (n = 54)	−17.43±1.11^3^	12.58±0.74^3^	56.6–68.1	0–3.4	31.3–41.6
	SM (n = 24)	−14.93±1.12^1^	13.33±0.92^1^	58.2–92.2	0–12.9	8.4–32.2
**2004**	NM (n = 54)	−19.02±0.85^4^	11.29±0.40^5^	27.2–35.5	0–3.6	64.0–70.3
	SM (n = 17)	−17.67±0.89^3^	11.91±0.54^4^	29.9–53.6	0–9.4	45.7–61.8

Stable isotope values for whole blood of Slender-billed Gull fledglings as a function of year (2001–2004) and breeding site (saltmines of Sanlúcar -SM-, the fish farm of Veta la Palma -FF- and natural marshes of Doñana -NM-). Pair-wise comparisons were done through Tamhane’s test procedure (overall alpha level of 0.05); identical superscript numbers indicate homogeneous sub-groups. Relative importance (95% Bayesian Credibility Intervals, BCI) of food from the three sites (SM, FF and NM) in the diet of fledglings hatched at different sites and in different years was inferred through a Bayesian double-isotope (δ^13^C and δ^15^N) isotopic mixing model (SIAR, [29]).

Based on previous information on feeding preferences of this species [11], [23], [24], fish from the natural marshes (Common Mummichogs *Fundulus heteroclitus* and Koi *Cyprinus carpio*) and from the fish farm (Eastern Mosquitofish *Gambusia holbrooki*), as well as brine-shrimps *Artemia* spp from the saltmines were collected during the 2004 breeding season for isotopic determination of main dietary resources. Once at the laboratory, all samples (i.e. fledglings’ blood and prey items) were frozen until further preparation for stable isotope analyses.

### Sample Preparation and Laboratory Analyses

All samples were dried in an oven at 60°C to constant mass and ground to a powder. Two aliquots were removed from each prey sample; one aliquot was immediately prepared for δ^15^N analysis, whereas the other underwent lipid extraction prior to δ^13^C analysis following the methods described at [25]. About 0.9–1 mg of each sample were combusted at 1020°C using a continuous-flow isotope-ratio mass spectrometry (CFIRMS) system consisting of a Carlo Erba 1500NC elemental analyzer interfaced with a Delta Plus XL mass spectrometer. Stable isotope ratios are expressed in the standard δ-notation (‰) relative to Vienna Pee Dee Belemnite (δ^13^C) and atmospheric N_2_ (δ^15^N). Replicate assays of laboratory standards (urea and shark cartilage) previously calibrated with international reference materials (IAEA-CH-6, IAEA-N-2 and USGS-40), and routinely inserted within the sampling sequence, indicated analytical measurement errors of ±0.1 ‰ for δ^13^C and ±0.2 ‰ for δ^15^N.

### Parameter Estimations and Statistical Testing

Estimations of the extent of marshland (see Figure 1) for the periods of interest were obtained by accessing all available cloud-free Landsat MSS, TM and ETM+ scenes for the Doñana region between 1983 and 2004 (190 scenes). Images were geometrically and radiometrically corrected, transformed into reflectance values using Pons and Solé-Sugrañes [26] method based on a dark object model, and normalized to a reference image using a set of pseudo-invariant areas [27]. Inundation rate was determined using mid-infrared band 5 (1.55–1.75 µm, TM and ETM+) and band 4 (0.8–1.1 µm, MSS) to produce final inundation masks based on pixels of 30×30 m (details in [27], [28]; examples in Figure 1). Since Landsat images were available approximately every 15 days (depending on cloud coverage), and because we were interested in obtaining integrative information on inundation levels for key periods comparable among years (see below), piecewise cubic Hermite interpolation of existing information was used to estimate the daily extent of the marshland for the time span included in this study.

To evaluate the influence of inter-annual fluctuations in the availability of natural resources on the reproductive performance of gulls at our study site, we obtained annual estimations of the extent of natural marshes for the entire breeding season (1^st^ April-31^th^ July), but also for the clutch production (1^st^ April–15^th^ May), incubation (15^th^ May–15^th^ June), and chick-rearing periods (15^th^ June–31^th^ July), by averaging interpolated daily data for the periods of interest. Estimated flooded areas were subsequently incorporated as explanatory variables in a stepwise regression to test their effects over gull reproductive success (estimated as the ratio between the total number of fledglings and the total number of breeding pairs). In this analysis we only incorporated data for the 1993–2004 period because of the lack of periodic and standardized data on population parameters before 1993 (see above).

Insights into the role of the two adjacent, anthropogenic systems as alternative feeding areas for gulls feeding chicks were obtained by investigating how availability of natural resources during the chick-rearing period affected the dietary composition of fledglings. Chick diets (split by year and breeding site) were inferred through a double-isotope (δ^13^C and δ^15^N), three endpoint (fish from the natural marshes and from the fish farm, and brine-shrimp from the saltmines) Bayesian mixing model (SIAR, [29]). Dietary endpoints included in these models were isotopically clustered (mean ± SD) by grouping potential prey from each foraging area. One-way ANOVA (with Welch’s correction to account for heteroscedasticity) and the Tamhane’s tests procedure (for post-hoc pair-wise analyses; [30]) were used for testing differences in the isotopic composition of dietary endpoints, which were subsequently adjusted to account for isotopic discrimination factors (ΔX) linking diet with consumers’ tissues (Δ^13^C: ∼1.1 ‰; Δ^15^N: ∼2.9 ‰; see [31]). The estimated extent of the marshland for the chick-rearing period was finally incorporated in a linear model to evaluate the influence of natural resource availability on the relative contribution of main dietary resources to the diet of fledglings. Complementary to our isotope approach, insights into the effect of natural resource availability on feeding habitat choice by gulls were obtained by investigating the relationship between estimated flooded areas at the natural marshes and the proportion of adult gulls (respect the total number of reproducing birds) observed at the saltmines (the main alternate foraging habitat, see Results). In particular, estimated proportions of gulls observed were fitted in a linear model in which flooded area and breeding stage (i.e. clutch production, incubation and chick-rearing) were included as covariate and fixed factor, respectively.

Information on resource use by gulls obtained throughout our isotope study was finally used to evaluated the effect of potential dietary shifts towards exploitation of alternate, anthropogenic resources on gulls’ breeding performance. Mean relative contributions of food from natural marshes derived from the mixing models were incorporated in a linear model to test the effect of this explanatory variable on reproductive success (split by year and breeding site) of our gull population. We also investigated the relationship between fledglings’ diet and body condition (estimated as the residuals of the linear regression of body mass, i.e. body weight, and body size, i.e. maximum head length, [32]) at the individual level through linear models in which individual body conditions was included as the response variable and raw δ^13^C and δ^15^N values for fledglings’ blood were incorporated as explanatory variables (provided that natural resources showed depleted isotopic values with respect those ascribed to man-made systems, see Results). To account for the dependence among fledglings raised at the same colony and during the same year, the combination of year and breeding site was included as a random factor. Analyses were done using MatLab 7.11 software (MathWorks Inc., Massachusetts, USA) for interpolations and SPSS 18.0 (SPSS Inc., Chicago, Illinois, USA) for statistical testing.

## Results

### Marshland Extent and Reproductive Success

Estimated average extent of the marshland strongly varied among years included in this study. Overall, the average marshland extent for the Slender-billed Gull breeding period ranged from extreme flood-years like 2004, when the average extent of the marshland was 15,049 ha, to extreme drought-years like 1995, with an average extent of flooded area of 151 ha (see Figure 2 for the temporal trend in the inundation levels). In particular, and accordingly to the flooding regime at the natural marshes (see above), chick-rearing matches up with the driest period, with average flooded areas ranging from 62 ha (1983) to 9,752 ha (1985), followed by the incubation (from 129 ha in 1995 and 17,478 ha in 1996) and the clutch-production period (from 226 ha in 1995 to 21,937 ha in 1996). Similarly, great inter-annual differences in gulls’ breeding performance were observed for this time-period. In particular, estimated reproductive successes ranged from ca. 0 (e.g. 1993 or 1994; values before 1993 were not considered) to ca. 1.7 chicks of fledgling age per nest (e.g. 1997 or 2004, see Figure 2), likely depending on the inundation levels at the marshland for the entire breeding season as suggested by the observed positive relationship between estimated flooded areas and reproductive success (selected model from the stepwise regression included the average extent of the natural marshes for the entire breeding period as the only explanatory variable, F_1, 10_ = 10.6, p = 0.01).

### Isotopic Interpretation and Dietary Changes

Significant differences were detected in the isotopic composition of prey from the three main foraging areas (F_Welch 2, 6.1_ = 22.9, p = 0.001 for δ^13^C and F_Welch 2, 6.5_ = 47.3, p<0.001 for δ^15^N, see Table 1 for pair-wise comparisons), thus allowing the use of mixing models to transform isotopic information into relative contributions of potential food resources to the diet of fledglings. In particular, brine-shrimp from the saltmines showed the highest δ^13^C values (mean ± SD: −17.02±0.48 ‰), followed by fish from the fish farm (−20.13±1.77 ‰) and from the natural marshes (−21.94±2.13 ‰). Regarding δ^15^N, the highest values were observed at the fish farm (14.29±1.38 ‰), followed by the saltmines (11.48±0.54 ‰) and the natural marshes (8.28±0.81 ‰). Dietary inferences derived from mixing models (Table 1) revealed that saltmines and natural marshes constituted the main foraging areas regardless of the year and breeding site considered (with average mean contributions of ca. 67% and 28%, respectively), whereas the dietary exploitation of the fish farm was relatively low (ca. 5%), although it is noteworthy its role as breeding site (Table 1 and Figure 2). Remarkably, estimated dietary compositions were similar among breeding sites within single years, whereas great inter-annual variations in the relative contribution of the two main dietary endpoints were detected throughout the 2001–2004 time-period (Table 1).

**Figure 3:**
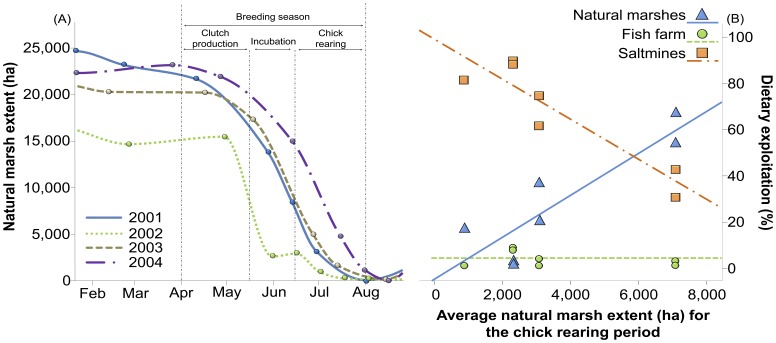
Linking natural resource availability with fledglings’ diet. (A) Temporal trends for the flooded areas (ha) at the natural marshes for the 2001–2004 period. Dots represent real flooded areas obtained from satellite images. (B) Relationship between the estimated flooded area at the natural marshes (ha) during the chick-rearing period and the estimated relative contribution of natural resources (blue), and resources from the fish farm (green) and the saltmines (orange) to the diet of Slender-billed Gull fledglings. Dots represent dietary estimates for fledglings hatched at different sites and in different years.

**Figure 4:**
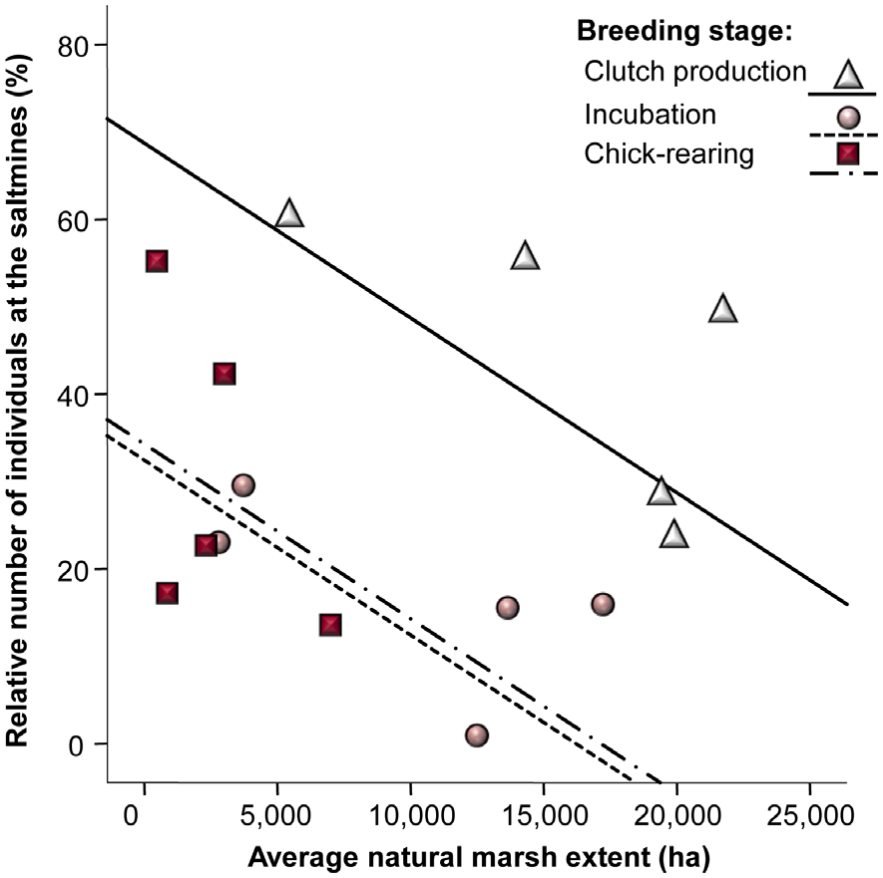
Linking natural resource availability with feeding habitat choice. Relationship between the estimated flooded area at the natural marshes (ha) during the clutch-production, the incubation and the chick-rearing period, and the proportion of adult gulls (i.e. respect the total number of reproducing birds) observed at the saltmines, i.e. the main alternate foraging habitat.

Inter-annual variations in the diet of fledglings strongly depended on the extent of the marshland during the chick-rearing period (Figure 3). In particular, increasing flooded areas at the natural marshes resulted in a higher dietary exploitation of this natural foraging habitat (F_1, 6_ = 19.8, p = 0.007), in contrast with the dietary contribution of saltmine resources (F_1, 6_ = 32.4, p = 0.002). Relative use of resources from the fish farm stood relatively constant regardless of observed variations in the extent of the natural marshland (F_1, 6_ = 0.53, p = 0.5). In agreement with obtained results from our isotope approach, direct evidence based on monthly censuses also suggested that natural resource availability had a role in determining feeding habitat choice by gulls (Figure 4). In particular, the proportion of gulls observed at the saltmines diminished as extent of the natural marshland increased (F_1, 15_ = 4.99, p = 0.047), a trend also found for the clutch-production, incubation and chick-rearing periods (F_2, 15_ = 0.36, p = 0.7, for the interaction between marshland extent and breeding stage).

**Figure 5:**
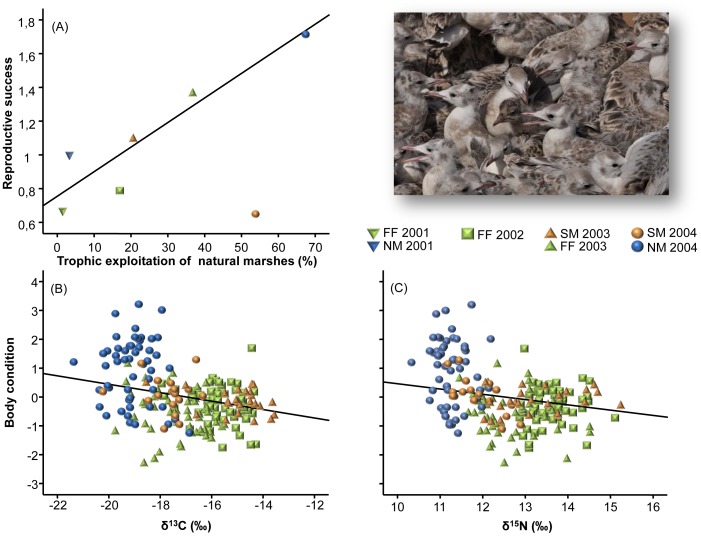
Linking diet with reproductive performance. (A) Relationship between estimated trophic exploitation of natural marshes (in %) by Slender-billed Gulls feeding chicks and the reproductive success of the population (split by breeding season and breeding site: saltmines of Sanlúcar -SM-, fish farm of Veta la Palma -FF- and natural marshes of Doñana -NM-). An unusual flooding episode caused a massive mortality of fledglings at SM 2004, and data on breeding success has been excluded from the regression. (B and C) relationships between raw isotopic values (δ^13^C and δ^15^N, in ‰) for fledglings’ blood and estimated body condition (residuals of the relationship body size-body weight). Morphometric data for the 2001 breeding season were not available, thus making impossible the estimation of fledglings’ body condition. Picture courtesy of M. Paracuellos.

Use of natural foods strongly affected the reproductive performance of the species (Figure 5). In particular, a greater use of natural marshes for food resulted in higher reproductive success (F_1, 5_ = 24.2, p = 0.008; note that Sanlúcar 2004 was excluded from the analysis since unusual rainfall levels partially flooded the colony and increased egg loss and chick mortality), and also in raising fledglings with better body condition (F_1,__106_ = 7.21, p = 0.008 and F_1,__106_ = 4.29, p = 0.041, for the observed negative relationship between fledglings’ body condition and raw δ^13^C and δ^15^N values, respectively; data were not available from 2001, when fledgling morphometrics were not recorded).

## Discussion

The connection between resource availability and population dynamics is likely mediated through the acquisition of resources and their allocation to one of the main components of animal fitness such as reproduction [33], [34]. Thus, studies aimed at comparing how animals acquire resources and their consequences for population breeding performance during periods of contrasting resource availability will be informed by an understanding of the impact of global environmental change on wild populations. Here, the Natural Area of Doñana provided an exceptional opportunity for such a study, because the observed inter-annual fluctuations in the extent of natural marshland forced wild populations breeding at this area to fine-tune each reproductive event to varying availability of natural and anthropogenically derived resources [18].

Slender-billed gulls breeding at the Natural Area of Doñana showed the greatest range in reproductive success (ranging from ca. 0 to 1.7 fledglings per breeding pairs) ever reported for the Northwestern Mediterranean (breeding success for the population breeding at the Ebro Delta during the period 1992–2001 ranged from ca. 0 to 1.25; [24]), with higher values for flood-years compared to drought years. Although these results cannot prove the causality of the relationship between the extent of natural marshland and the breeding performance of this gull population, similar results have been reported for other wild species inhabiting this area (e.g. Black-crowned Night Heron *Nycticorax nycticorax*, Cattle Egret *Bubulcus ibis*, Little Egret *Egretta garzetta*, or black kite *Milvus migrans*; [18], [35]). Thus, the availability of food from the natural marshes seems to play an important role in determining the reproductive performance of species breeding here.

Food availability has been described previously as a major factor influencing the reproductive output of wild populations [36]–[39]. However, few studies have unequivocally demonstrated the constraining effect of food limitation on population reproductive performance [40], [41]. The main reason for this appears to be the difficulty of monitoring variation in feeding preferences [41]. Indeed, investigating the relationship between food-abundances and reproductive performance is challenging, since individuals can switch to alternative resources when their staple prey becomes scarce, thus hampering rigorous analysis of the processes constraining food availability (see [18], [41]). In this regard, stable isotope analysis has developed into a powerful tool for monitoring spatio-temporal variations in the diet of target populations likely associated with changes in resource availabilities (e.g. [42]), thus allowing quantitative inferences on the relationship between natural resource availability, diet-choice and the consequential effect on the reproductive performance of wild populations.

A common assumption of all retrospective isotopic studies is that isotopic baselines have either not changed through time or that such changes can be accounted for (see [43], and references therein). In our study, isotopic data for prey were only available for 2004, and so we cannot completely rule out the possibility that observed inter-annual variations in dietary estimates were caused by temporal variations in the isotopic composition of main prey types. However, several arguments suggest that this hypothesis is an unlikely explanation for our observed temporal trends. Firstly, temporal shifts in the isotopic composition of particular food resources are expected to be small compared to the considerable isotopic variations caused by changes in bird diets and foraging locations (see [42]–[44]). Secondly, observed relationships between marshland extent and dietary estimates clearly agree with information obtained from direct observations on feeding habitat choice by gulls, but also with observed relationship between marshland extent and reproductive success for the 1993–2004 period (provided that dietary exploitation of natural foods likely resulted in higher reproductive success). Our information on resource use by gulls therefore suggested that, when available, individuals apparently tended to prefer fish from natural marshes, whereas saltmines were the main alternative foraging area. In contrast, the minor role of the fish farm as a feeding area was noteworthy, a fact that could well be the consequence of the several mechanisms that have been implemented at this system to avoid fish predation by avian species (e.g. ponds with the highest fish densities [ca. 0.15% of the fish farm pond area] are covered with a mesh).

Brine-shrimp is a low-quality food for birds because of its low energy content [45], difficulties inherent in their consumption (i.e. the low rate of intake per unit of time [46]), and the associated energy costs related to salt excretion [46]–[48]. Thus, Slender-billed Gulls could tend to maximize fledgling survival, and, in turn, their breeding performance, by proactively biasing their diets towards consumption of a resource with a higher energy value, such as fish [49], whenever this resource was available for the population. Indeed, the relative contribution of fish to the diet of chicks has previously proved to positively affect the breeding success of other gull species [50]–[53]. This was in agreement with our inferred positive relationship between relative consumption by gulls of fish from the marshland and estimated reproductive output (reproductive success and fledglings’ body condition).

In addition to inter-annual variations in the availability of natural food resources as mediated by flooding, varying predation pressure and/or inter-specific competition for food may also have a role in explaining observed temporal pattern in gulls’ reproductive output. Based on historical records provided by the Monitoring Team of Natural Processes, predation episodes were sporadic (only observed during the 1985, 1993, 1999 and 2000 breeding periods) and mostly affected individuals breeding at the natural marshes and the saltmines, which are particularly susceptible to be affected by terrestrial predators. Because of the sporadic nature of these episodes, predation would be expected to have a minor role in explaining the long-term pattern in the estimated reproductive output of our gull population. However, these episodes could partially explain variations between natural resource availability and gulls’ reproductive performance. For example, predation by rats (*Rattus norvegicus*) during the 1985 breeding period, when natural resources availability was relatively high (see Figure 2), caused the total failure of the gull population breeding at the natural marshes. On the other hand, and similar to Slender-billed Gulls, most of the other waterbird species inhabiting this area alternate their use of natural and man-made habitats (e.g. [17]). Variations in the availability of natural food resources may result, therefore, in varying inter-specific competition for resources ascribed to artificial systems, thus having an additive effect on the observed relationship between natural resource availability and gulls’ feeding preferences and reproductive output.

In conclusion, higher energy flux to chicks during flood years, due to an increase in the nutritional quality of their diet, is the more likely explanation for the observed positive relationship between the relative contribution of natural resources to the diet of fledglings and reproductive output (i.e. breeding success and fledglings’ body condition). However, the actual role of natural resource availability in constraining gull reproduction entails assessing the effect of feeding habitat choice by gulls on other fitness-related parameters, such as the likelihood of laying eggs or skipping reproduction, the number and size of eggs laid or the rate of hatching success (provided that adults segregate food for themselves from that delivered to their chicks [15]). In this regard, information derived from direct observations suggested that the availability of natural resources was also a relevant factor affecting adult gull diet, since the relationship between marshland extent and the proportion of birds feeding at the saltmines was also observed for the clutch-production and the incubation period. That the most important factor affecting reproductive success was the average extent of the natural marshes for the entire breeding period suggested that natural resources availability likely affected other reproductive parameters different from chick survival and fledgling body condition such as egg production and hatching success.

The extent of natural habitats available for wild species is currently being lost at ever increasing rates as a result of human activities [54]–[56]. So, there is an ongoing need to understand how wild populations adapt to the new circumstances and, particularly, the potential role of several alternative systems, such as fish farms or saltmines, as buffer areas against loss or degradation of natural feeding habitats. The benefit for wildlife of these anthropogenic structures as alternative breeding/feeding sites is undeniable as they provide individuals with a relatively safe and stable environments (see [57]). Indeed, since 1992, the fish farm of Veta la Palma has been the preferred breeding site for our Slender-billed Gull population (see Figure 2; see also [58]), and this artificial system may have played an important role in the exponential growth that this population has undergone during the last two decades. Similarly, these man-made systems have been previously considered as suitable alternative feeding areas for wild populations [9]. Here, we do not question the importance of these structures as alternative foraging habitats for free-living animals, but stress the fact that, at least for some species, they cannot completely compensate for loss of natural resources. We suggest our methodology could be extended to a large suite of natural communities that are potentially threatened as a result of habitat loss, thus providing a useful framework for management and conservation purposes.
